# Continuous Affinity Purification of Adeno-Associated Virus Using Periodic Counter-Current Chromatography

**DOI:** 10.3390/pharmaceutics14071346

**Published:** 2022-06-25

**Authors:** João P. Mendes, Magnus Bergman, Anita Solbrand, Cristina Peixoto, Manuel J. T. Carrondo, Ricardo J. S. Silva

**Affiliations:** 1 iBET, Instituto de Biologia Experimental e Tecnológica, Apartado 12, 2780-901 Oeiras, Portugal; jmendes@ibet.pt (J.P.M.); peixoto@ibet.pt (C.P.); mjtc@ibet.pt (M.J.T.C.); 2 ITQB NOVA, Instituto de Tecnologia Química e Biológica António Xavier, Universidade Nova de Lisboa, Av. da República, 2780-157 Oeiras, Portugal; 3 Cytiva, 751 84 Uppsala, Sweden; magnus.bergman@cytiva.com (M.B.); anita.solbrand@cytiva.com (A.S.)

**Keywords:** adeno-associated virus, affinity purification, continuous processing, gene therapy, periodic counter-current chromatography

## Abstract

Replacing batch unit operations of biopharmaceuticals by continuous manufacturing is a maturing concept, with periodic counter-current chromatography (PCC) favoured to replace batch chromatography. Continuous affinity capture of adeno-associated virus (AAV) using PCC has the potential to cope with the high doses required for AAV therapies thanks to its inherent high throughput. The implementation of continuous AAV affinity capture using a four-column PCC process is described herein. First, elution buffer screening was used to optimize virus recovery. Second, breakthrough curves were generated and described using a mechanistic model, which was later used to characterize the loading zone of the PCC. The experimental runs achieved a stable cyclic steady state yielding virus recoveries in line with the optimized batch process (>82%), with almost a three-fold improvement in productivity. The PCC affinity capture process developed here can bolster further improvements to process economics and manufacturing footprint, thereby contributing to the integrated continuous manufacturing concept.

## 1. Introduction

Adeno-associated viruses have gained attention as a gene delivery vector thanks to their safety profile, long term expression, ability to transduce differentiated cells, and the vast repertoire of available serotypes [1,2]. Depending on the medical condition being treated, vector requirements for therapies under a clinical development scenario can reach $10^{14}$ VG/kg [3], requiring substantial manufacturing operation improvements for the successful development of these therapies.

Downstream purification of viral vectors, in particular recombinant adeno-associated virus (AAV), has been extensively discussed in the literature [4,5,6]. Density gradient ultracentrifugation with cesium chloride (CsCl) or iodixanol is one of the most recurrent methods for laboratorial AAV purification. However, as the product moves through the different clinical stages the need for scalable purification technologies compatible with good manufacturing practices (GMP) increases. Chromatography-based purification can be easily adapted for large-scale production. The use of different chromatography modes for AAV purification using different strategies, is well described in the literature [7,8,9], including such methods as size-exclusion, ion exchange, and affinity chromatography (AC). More recently, the development of AC media with ligands against several AAV serotypes has paved the way for more efficient purification strategies. This follows mAb purification practices, where the early use of Protein A chromatography in the manufacturing process was able to reduces the overall number of purification steps, thus leading to increased downstream processing production rates.

**Figure 1:**
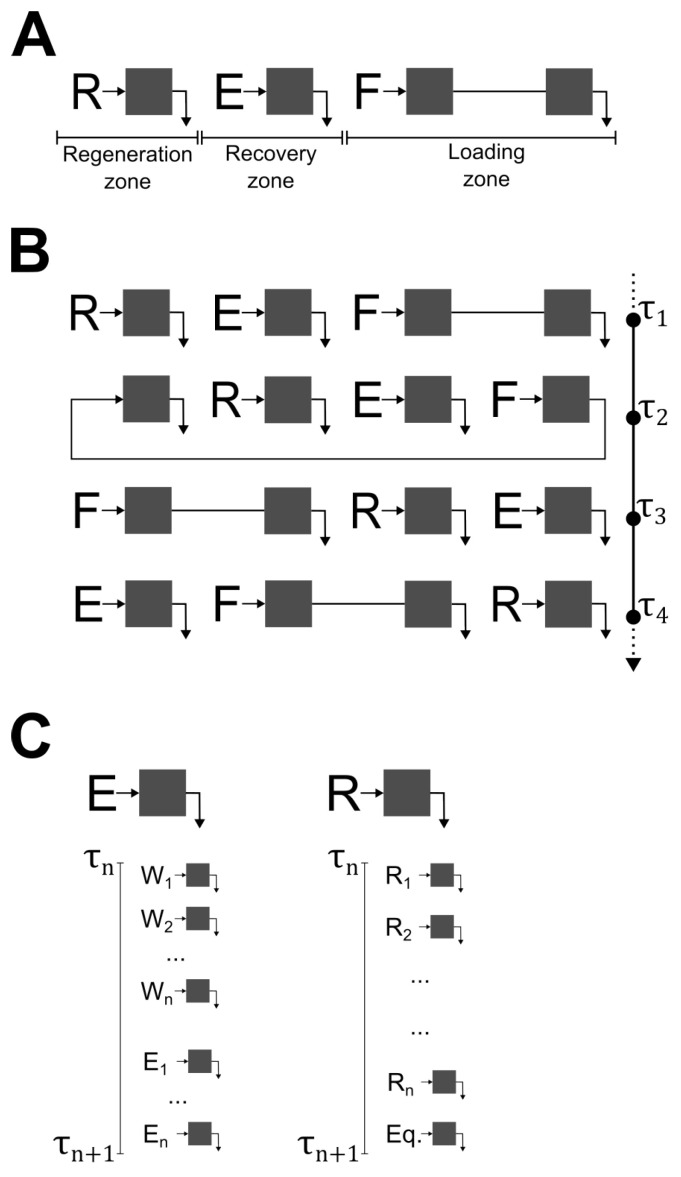
Schematic of a four-column periodic counter-current process. (**A**) Chromatography zones: a recovery zone for product elution, a regeneration zone for cleaning in place and re-equilibration, and a loading zone with two columns connected in series. (**B**) Operating cycle, composed of four switching intervals (*τ*) of equal duration. (**C**) Description of elution and regeneration zones and possible substeps; W${}_{1}$ to W${}_{n}$, E${}_{1}$ to E${}_{n}$, R${}_{1}$ to R${}_{n}$, and Eq. denote the several steps for washing, elution, column regeneration, and column equilibration, respectively.

Over the last two decades, several research groups have reported on improvements to the chromatographic process realized by replacing batch operation with continuous processing alternatives. This has been demonstrated for the purification of recombinant proteins [10,11], mAbs [12,13], mAb fragments [14], viruses [15,16,17,18,19], and exosomes [20], for which different multi-column chromatography processes have been utilized. These processes operate in continuous or semi-continuous mode with distinct column configurations, enabling significant improvements regarding productivity, resin utilization, and buffer consumption while keeping product quality and yield at least similar to that obtained in batch processes. Periodic counter-current chromatography (PCC) technology was designed by Cytiva for continuous application. This technology is based on customization of the ÄKTA^™^ platform, allowing for several simultaneous operations in a train of three or four columns placed in series, ultimately enabling a continuous product load. Generally, a four-column PCC (4C-PCC) process, such as that depicted in Figure 1A, is composed of three zones: a recovery zone where the purified product is eluted; a regeneration zone where the adsorptive beds are prepared for the next loading step (after cleaning in place and re-equilibration); and a loading zone with two columns connected in series. A basic cycle of a 4C-PCC is composed of four switching intervals of equal duration (*τ*).

During a cycle, all columns are subject to the same sequence of operations, as seen in Figure 1B. Briefly, the cycle starts with the third column being loaded. In order to maximize resin utilization, the effluent of this column is sent to the fourth column of the train. This allows for an increase in the saturation of the third column and capture of the mass transfer zone on the fourth column, avoiding loss of product in waste effluents. Simultaneous with this operation, the first column is regenerated, while the second column, loaded in a previous step, is subject to wash and product elution (Figure 1C). After all of these operations are concluded, the inlet and outlet ports move one column in the direction of fluid flow, similar to a classical simulated moving bed process. Feed continuity is dependent on a design constraint. In order to achieve synchronized periodic operation, the load period must be equal to or longer than the period required for product recovery and regeneration operations.

Several advances in integrated and continuous bioprocessing technologies have been reported and reviewed in recent years, especially for mAbs [21,22,23]. While the focus has mainly been directed toward establishing continuous production bioreactors, it is necessary to extend intensified processing to downstream unit operations as well. In the current study, a PCC process for affinity capture of AAV2 is established and assessed using real feed conditions. Here, the optimization of binding and elution conditions were carried out using small-scale models and Design of Experiments. Breakthrough curve analysis was performed using a mechanistic mathematical model of the loading zone to help design the PCC process. Although the true potential of PCC use can only be fully realized upon integration with other continuous operating units (upstream and downstream of PCC), the results of this proof of concept represent a step forward in the development and implementation of integrated and continuous bioprocessing for virus-based gene therapy products.

## 2. Materials and Methods

### 2.1. AAV Production

Human Embryonic Kidney 293T (HEK 293T) adherent cells purchased from ATCC (ATCC-CRL-3216) were cultured and expanded using DMEM medium (Thermo Fisher Scientific, Waltham, MA, USA), supplemented with 4.5 g L${}^{-1}$ of glucose (Merck, Darmstadt, Germany), 2 mM of glutamine (Thermo Fisher Scientific), and 10% (*v*/*v*) FBS (Thermo Fisher Scientific) under a humidified atmosphere of 7% CO${}_{2}$ in air at 37 °C. Cell factories with ten layers (Thermo Fisher Scientific) were inoculated at 8$\;\times \;10^{4}$ cells cm${}^{-2}$. Twenty-four hours after inoculation, the cells were transfected with a plasmid cocktail mix in serum-free DMEM medium (Thermo Fisher Scientific) supplemented with 4.5 g L${}^{-1}$ of glucose (Merck), 2 mM of glutamine (Thermo Fisher Scientific) making for 10% of total cell factory volume. The cocktail mix contained a 5:5:1 molar ratio of pHelper:pAAV-RC2:pAAV-GFP with a ratio of 2.9 μg of total plasmid DNA per million cells at the transfection moment. The transfection reagent (PEI MAX, PolySciences) to plasmid ratio was 1:2. The medium was exchanged 24 h post-transfection to a serum-free DMEM medium (Thermo Fisher Scientific) supplemented with 4.5 g L${}^{-1}$ of glucose (Merck) and 2 mM of glutamine (Thermo Fisher Scientific).

Cell harvest was performed at 72 h post-transfection using TrypLE Select (Thermo Fisher Scientific) neutralized with the serum-free DMEM medium described. Following harvest, the cells were centrifuged for 40 min at 300× *g* at 4 °C and resuspended in lysis buffer composed of 50 mM Tris, 0.1% Triton X-100 (Sigma Aldrich, St. Louis, MO, USA), and 2 mM of MgCl${}_{2}$.

Cell lysis was carried out for 1 h at 37 °C with 100 Units mL${}^{-1}$ of Benzonase (Merck, Darmstadt, Germany) with mild agitation. To stabilize the virus in solution, MgSO${}_{4}$ and NaCl were added to make up a final concentration of 37.5 mM and 400 mM, respectively, followed by incubation for 30 min at 37 °C with the same agitation rate.

The treated harvest was clarified using a 5.0 µm ULTA Capsule GF (Cytiva, Uppsala, Sweden) followed by a 0.8/0.2 µm Sartopore 2 XLG (Sartorius Stedim Biotech, Göttingen, Germany). The filters were previously rinsed with 20 mM Tris and 500 mM NaCl at pH 8.

### 2.2. Screening of Elution Buffer

Capto^™^ AVB in slurry format (Cytiva, Uppsala, Sweden) was used for elution buffer screening using a full factorial Design of Experiments. The factors used were the concentration of NaCl and L-Arginine concentration (0–500 mM) supplementing an elution buffer composed of 50 mM of citrate, pH 3.5. The UV${}_{280\;\mathrm{nm}}$ of the eluates was used as response. Three center points were considered.

The resin slurry (10 mL) was centrifuged at 300× *g* for 10 min. The supernatant was removed and replaced by 20 mM Tris and 500 mM of NaCl, pH 8.0. The resin was re-suspended, left in contact with the buffer for 10 min, and subjected to two new cycles of centrifugation and buffer exchange. The slurry was than centrifuged again and the buffer was removed and replaced by 10 mL of clarified AAV sample. After 1 h of incubation, the slurry was re-suspended by pippeting and dispensed in fractions of 1 mL. Each fraction was centrifuged and washed with 1 mL of 20 mM Tris, pH 8.0. This procedure was repeated three times. Finally, after a new round of centrifugation, the different eppendorfs containing Capto AVB resin and adsorbed AAV were eluted by applying 0.2 mL of the different elution buffer composition dictated by the DoE, then left to incubate for 30 min. The supernatant was recovered and analysed by UV${}_{280\;\mathrm{nm}}$ in a NanoDrop (Thermo Fisher Scientific), using the elution buffer as a blank.

### 2.3. Batch Chromatography and Frontal Experiments

Elution buffer optimization runs and frontal experiments for determination of the breakthrough curves were carried out in an ÄKTA^™^ avant 150 (Cytiva) equipped with a built-in fraction collector. Pre-packed OPUS^®^ MiniChrom^™^ columns (Repligen, Waltham, MA, USA) with 0.2 mL of Capto AVB (Cytiva) were used. Prior to use, each column was regenerated with 50 CV of 10 mM of NaOH, followed by a cycle composed of 50 CV of equilibration buffer, 50 CV of elution buffer, and finally 50 CV of equilibration buffer.

### 2.4. 4C-PCC Experiments

An ÄKTA pcc 75 system was operated in four-column mode (4C-PCC) using pre-packed OPUS MiniChrom columns with 0.2 mL of Capto AVB. The equilibration buffer was composed of 20 mM Tris and 500 mM NaCl, pH 8.0, the wash buffer of 20 mM Tris, pH 8.0, and the elution buffer of 50 mM citrate, 500 mM NaCl, and 350 mM L-arginine, pH 3.5. Regeneration was performed using 10 mM NaOH. Eluted pools were neutralized using 1 M Tris, pH 8.0 (10% of the pool volume). The 4C-PCC process used a sample pump to dispense 175 column volumes (CV) of clarified AAV2 sample per cycle. System pump A was used for elution procedures; washing was carried out with 25 CV of equilibration buffer followed by 25 CV of elution buffer and 20 CV of equilibration buffer. System pump B was used in the regeneration zone; 20 CV of 10 mM NaOH was injected, followed by 20 CV of equilibration buffer.

### 2.5. Analytical Quantification Methods

#### 2.5.1. Total Particle Quantification

The determination of total AAV particle concentration (TP) was carried out with a conformational AAV2 ELISA assay (Progen Biotechnik GMBH, Heidelberg, Germany) according to the manufacturer’s instructions. The reference curve was built from 2.3 × 10${}^{9}$ TP mL${}^{-1}$ with serial dilutions to 1.8 × 10${}^{7}$ TP mL${}^{-1}$. The absorbance was quantified at 450 nm on an Infinite PRO NanoQuant (Tecan, Männedorf, Switzerland) microplate multimode reader using a clear 96-plate well provided in the kit. The samples were applied at multiple dilutions.

#### 2.5.2. Genome Copies Quantification

DNA extraction was performed according to the instructions in the High Pure Viral Nucleic Acid Kit (Roche, Basel, Switzerland) manual, and was followed by real-time PCR. Determination of the number of viral DNA copies was performed using a LightCycler system (Roche Diagnostic) with probe (5${}^{\prime }$-TTGCCGTCCTCCTTGAAGTCGAT-3${}^{\prime }$). The reference standard used was transgene GFP plasmid with serial dilutions from 1 × 10${}^{8}$ copies µL${}^{-1}$ and specially-designed primers for the genome (forward primer 5${}^{\prime }$-GAACCGCATCGAGCTGAA-3${}^{\prime }$ and reverse primer 5${}^{\prime }$-TGCTTGTCGGCCATGATATAG-3${}^{\prime }$).

#### 2.5.3. Total Protein and ds-DNA

The protein and ds-DNA content were assessed with specific assays according to the manufactuer’s instruction of each. The total protein content was quantified using a BCA Protein Assay Kit (Thermo Fisher Scientific) and total ds-DNA was quantified with a Quant-iT^™^ Picogreen^®^ dsDNA assay kit (P7589, Invitrogen^™^, Waltham, MA, USA). Samples were applied in serial dilutions and reference material was applied in duplicate. Absorbance with respect to protein quantification and fluorescence with respect to ds-DNA quantification were measured with an Infinite PRO NanoQuant (Tecan) microplate reader.

#### 2.5.4. SDS-PAGE and Western Blot

The protein profile analysis was carried out in NuPage gradient pre-cast gels (Invitrogen) after protein denaturation. The gels were run for 60 min at a constant voltage of 180 V and stained with Coomassie Instant Blue (Expedeon Ltd., Cambridge, MA, USA) following membrane transfer. The iBlot system (Invitrogen) was used to transfer the AAV proteins to the PVDF membrane. The membrane was blocked in Tris-buffered saline with 0.1% (*w*/*v*) of Tween 20 with 5% (*w*/*v*) milk for 1 h. Then, immunostaining was performed overnight using anti-AAV VP1, VP2, and VP3 mouse mono IgG1 clone B1 (Progen Biotechnik GMBH, Heidelberg, Germany) primary antibody. Afterwards, the membranes were washed and incubated with the secondary antibody, anti-Mouse IgG (γ-chain specific) (Merck) for 1 h. The proteins were revealed using alkaline phosphatase.

#### 2.5.5. Transmission Electron Microscopy

Transmission electron microscopy (TEM) was performed in order to assess the presence and quality of AAVs. A volume of 5 µL of sample was adsorbed onto a Formvar-coated 150 mesh copper grid from Veco (Science Services) for 2 min. The grid was washed with sterile water and a solution of 2% uranyl acetate was added for 2 min and left to dry at room temperature. The image was taken with a Hitachi H-7650 120 kV electron microscope (Hitachi High-Technologies Corporation, Tokyo, Japan).

### 2.6. Chromatography Model

The transport of virus along the axial dimension (*z*) of a chromatographic column can be described using the following material balance [24]:

(1) $$\begin{array}{c} \epsilon \frac{dc}{dt}+\left(1-\epsilon \right)\frac{dq}{dt}+v\frac{\partial c}{\partial z}=D_{ax}\frac{\partial^{2}c}{\partial z^{2}} \end{array}$$

where *c* [TP mL${}^{-1}$] and *q* [TP mL${}^{-1}$] are the concentration of virus in the liquid and stationary phases, respectively, $D_{ax}$ [cm${}^{2}$ min${}^{-1}$] is the dispersion coefficient, ϵ is the porosity of the column, and *v* [cm min${}^{-1}$] is the interstitial velocity. The dispersion coefficient is correlated to the flow by a Peclet number ($Pe=\frac{uL}{D_{ax}}$, with *L* [cm] being column height). Equation (1) is subject to the following boundary conditions:

(2) $$\begin{array}{cc} \frac{\partial c}{\partial z}=\frac{v}{D_{ax}}\left(c-c^{in}\right), & \;\mathrm{for}\;z\;=\;0 \end{array}$$

(3) $$\begin{array}{cc} \;\frac{\partial c}{\partial z}=0, & \;\mathrm{for}\;z\;=\;1\; \end{array}$$

where $c^{in}$ is the virus concentration at the column inlet. The adsorption of virus to the particle surface is described by a kinetic form of the Langmuir adsorption isotherm, as described in Equation (4), with $k_{ads}$, $k_{des}$ the adsorption and desorption coefficients and $q^{sat}$ the maximum adsorption capacity.

(4) $$\begin{array}{c} \frac{dq}{dt}=k_{ads}c\left(q^{sat}-q\right)-k_{des}q \end{array}$$

The nonlinear chromatography model was solved numerically using a full discretization in both the time and space coordinates.

## 3. Results

### 3.1. Optimization of Elution Buffer

**Figure 2:**
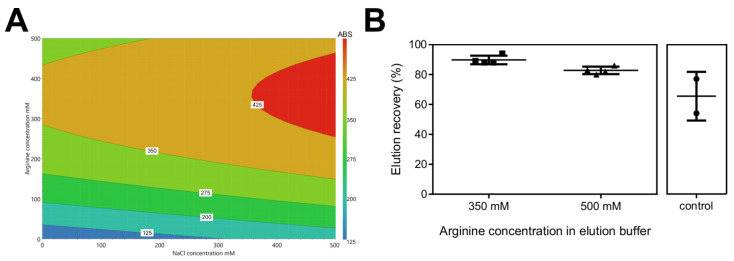
Optimization of elution buffer. (**A**) Response contour plot (UV${}_{280}$ of eluate pools); experimental matrix and summary of fit are reported in Supplementary Table S1 and Figure S1, respectively. (**B**) Validation experiments performed in column format: a clarified AAV2 sample (35 mL) was injected into a 1 mL Capto AVB column; the composition of the elution buffers used was 50 mM citrate and 500 mM of NaCl, pH 3.5, supplemented with 350 or 500 mM of arginine, batch control was performed by injecting a clarified AAV2 sample (35 mL) into a 1 mL Capto AVB column, and the elution buffer was 50 mM citrate, pH 3.5.

In order to optimize AAV2 elution recovery, a DoE screening study was performed. The elution buffer composition (50 mM citrate, pH 3.5) was adjusted by supplementing NaCl and arginine according to the experimental design matrix. Figure 2A depicts the response (UV${}_{280}$ of eluate pools) contour plot. The response is mildly affected by NaCl within the examined range. In comparison, a local maximum is observed for concentrations of arginine in the range of 275 to 475 mM, along with NaCl concentrations above 375 mM. To confirm this screening, we carried out two sets of column experiments with different arginine concentration (350 and 500 mM) at a fixed NaCl concentration of 500 mM. The elution pools were analysed by ELISA, and both sets were characterized using the calculated elution recovery. As seen in Figure 2B, 500 mM of arginine yields a lower AAV elution recovery average in comparison to the 350 mM concentration (82.7 vs. 89.8%). This is in line with the prediction from the DoE response contour plot, and is higher than the average of the control experiments (65.5%).

### 3.2. Breakthrough Curves

In order to understand the behaviour of the breakthrough curves under different operating conditions, we carried out frontal experiments using clarified samples with different AAV2 concentrations (low-level: 6.40 $\times \;10^{11}$ TP mL${}^{-1}$, high-level (average): 2.10 $\times \;10^{12}$ TP mL${}^{-1}$) and flow-rates (low-level: 0.33 mL min${}^{-1}$, high-level: 0.51 mL min${}^{-1}$). The results show that higher flow rates promote earlier breakthrough of the chromatography beds for both levels of AAV2 concentrations used.

**Figure 3:**
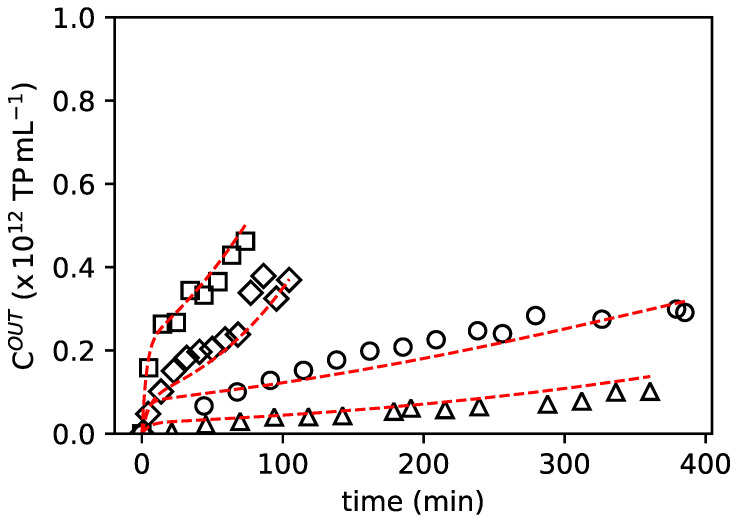
Fitting of breakthrough experiments with low-level of feed concentration: ○: c${}_{\mathrm{FEED}}=6.40\times 10^{11}$ TP mL${}^{-1}$ and Q${}_{\mathrm{FEED}}=0.51$ mL min${}^{-1}$, Δ: c${}_{\mathrm{FEED}}=6.40\times 10^{11}$ TP mL${}^{-1}$ and Q${}_{\mathrm{FEED}}=0.33$ mL min${}^{-1}$. With high-level of feed concentration: ◇: c${}_{\mathrm{FEED}}=2.21\times 10^{12}$ TP mL${}^{-1}$ and Q${}_{\mathrm{FEED}}=0.33$ mL min${}^{-1}$, □: c${}_{\mathrm{FEED}}=1.90\times 10^{12}$ TP mL${}^{-1}$ and Q${}_{\mathrm{FEED}}=0.51$ mL min${}^{-1}$. The red line represents chromatography model fitting.

The recovered samples during column loading were analysed using ELISA. The generated breakthrough curves (reported in Figure 3) were fitted using the chromatography model referenced in Section 2.6. The adsorption parameters (Table 1) were estimated using the inverse method of chromatography [24], where calculated breakthrough profiles are fitted to experimental ones obtained from frontal experiments performed at different flow rates and virus feed concentrations. As seen in Figure 3A,B, the model predicts experimental data reasonably well, with deviations observed only in the case of lower AAV2 feed concentrations.

### 3.3. Chromatography Model of 4C-PCC Loading Zone

The axial concentration profiles of the loading zone of the 4C-PCC process were simulated at the end of each switching interval (*τ*) of the first cycle using the fitting parameters reported in Table 1. The simulations were performed for an arbitrary loading of 200 CV at two different flow-rates (0.33 and 0.51 mL min${}^{-1}$). A reference concentration (c${}_{ref}$) of 2 × 10${}^{12}$ TP mL${}^{-1}$ was considered to be representative of the titers obtained in clarified AAV2 samples.

**Figure 4:**
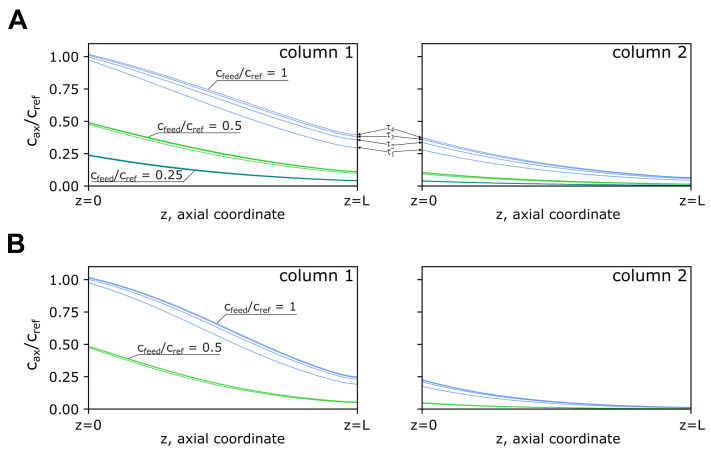
Axial concentration profiles of the 4C-PCC loading zone: simulations using a load flow-rate of (**A**) 0.51 mL min${}^{-1}$ and (**B**) 0.33 mL min${}^{-1}$; c${}_{ax}$, c${}_{ref}$, and c${}_{feed}$ are the axial, reference, and feed concentrations, respectively; c${}_{feed}$ used 2, 1, and 0.5 × 10${}^{12}$ TP mL${}^{-1}$, while the axial concentration profiles of the loading zone were calculated at the end of each switching interval (*τ*) composing the first cycle of the 4C-PCC process. The scenario of c${}_{ref}$/c${}_{feed}=0.25$ was not represented for the flow rate of 0.33 mL min${}^{-1}$, as the axial profile did not reach the column 1 outlet.

A closer look at Figure 4 reveals that low saturation of the loading zone is obtained with lower feed concentrations (c${}_{feed}$/c${}_{ref}<1$) (Figure 4A,B). The same is observed in Figure 4B for a flow-rate of 0.33 mL min${}^{-1}$ and independent of the feed concentration. The setting using the highest flow-rate (0.51 mL min${}^{-1}$) and feed concentration (c${}_{feed}$/c${}_{ref}=1$) provides a higher bed saturation; however, the outlet concentration of the second column of the loading zone reaches approximately 7% of the feed concentration at the end of the first cycle (*τ*_4_). A reduction of the loading volume to 175 CV using the highest setting for flow rate and feed concentration was chosen in order to minimize losses. Under this scenario, the outlet concentration is reduced to approximately 1% of the feed concentration at the end of the first cycle. A load volume of 175 CV and a flow-rate of 0.51 mL min${}^{-1}$ for the 4C-PCC processes was utilized.

### 3.4. 4C-PCC Affinity Capture Process

**Figure 5:**
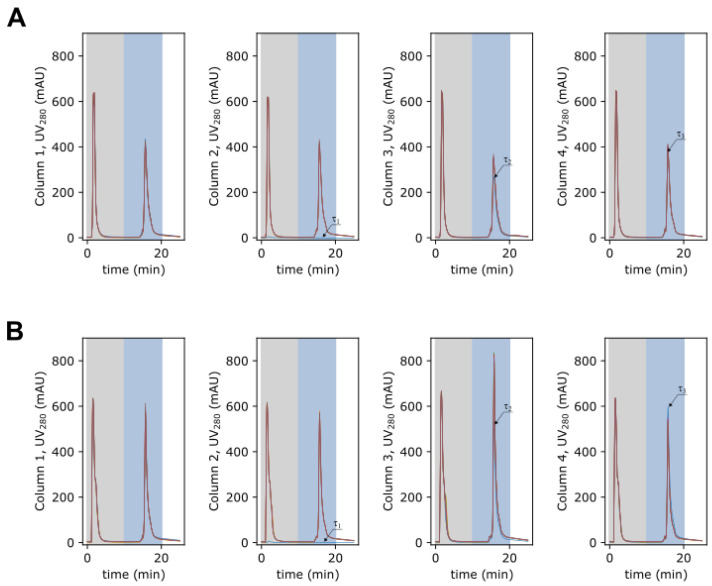
Chromatograms of PCC: run 1 (**A**) and run 2 (**B**); detail of the initial 25 min of each switching interval. The gray zone marks column wash and light blue marks elution.

**Figure 6:**
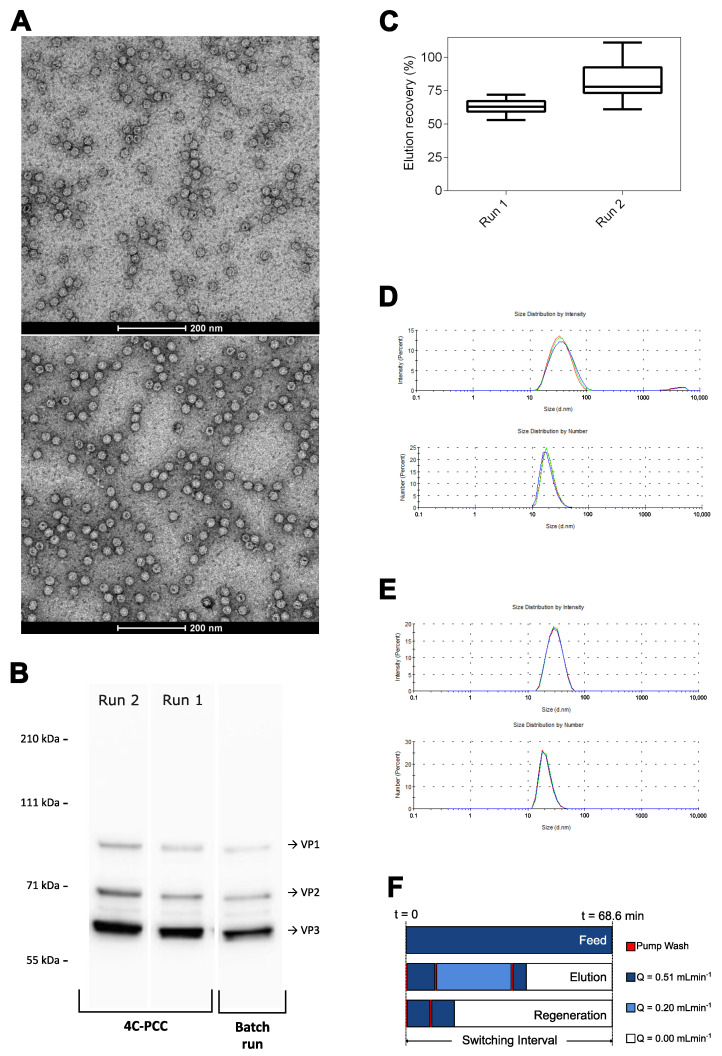
Characterization of the eluted pools from PCC runs 1 and 2: (**A**) transmission electron microscopy (top, PCC run 1; bottom, PCC run 2); (**B**) Western blot targeting AAV capsid proteins; (**C**) elution recovery, calculated using ELISA. Dynamic light scattering analysis (n = 3) of (**D**) PCC run 1 and (**E**) PCC run 2; for both runs, the top distributions are intensity-weighted and the bottom distribution are number-weighted. (**F**) Occupation of sample, system A and system B pumps in the PCC process.

In order to evaluate the potential of using periodic counter-current chromatography for affinity capture of AAV, two processes using a 4C-PCC were performed. After column equilibration, the PCC process starts with the regeneration zone in column 1, the recovery zone placed in column 2, and the loading zone in columns 3 and 4. The gray rectangles in the chromatograms in Figure 5 indicate the period of column washing, while the light blue indicates AAV elution. The chromatograms detailed in Figure 5A,B report the overlapping of the initial 25 min of each switching interval as grouped by the column placed in the elution zone. Here, the material accumulation is visible for the first cycle. No differences are observed among the elution peaks over the remaining cycles after this initial period. The UV${}_{280}$ profiles obtained at the outlet of the elution zone over the six cycles performed in each run (A and B) are reported in Supplementary Figure S2. For both processes, it can be observed that a cyclic pattern is reached. The characterization of the eluted pools is reported in Figure 6. Transmission electron microscopy was used to study the morphology of eluted AAV after negative staining. Representative images of the PCC processes are shown in Figure 6A, all at the same magnification. The intact AAV particles show an icosahedral symmetry or spherical shape with a diameter of approximately 20–30 nm. Western blots (Figure 6B) using monoclonal antibody targeting AAV capsid proteins detected the main AAV capsid proteins (VP1, VP2, and VP3) in both PCC experiments. Elution recovery was calculated using ELISA and is reported in Figure 6C. The first run had an average recovery of 63.2%, compared to 82.8% for the second run. Dynamic light scattering was used to assess particle size distribution; Figure 6D and E show PCC runs 1 and 2, respectively. The intensity-weighted distribution reveals minor differences among the two process elution pools. For the first run, an average dimension of 32.60 nm with a polidispersity index of 0.234 is obtained and, two populations are observed. For the second run, an average dimension of 26.87 nm with a polidispersity index of 0.144 is obtained. In this run, only one population is observed. The number-weighted distributions show that distributions in the range of 10–100 nm to be predominant. Concomitant with the characterization provided in Figure 6, the elution pools were analysed for ds-DNA, total protein, and VG. The first run yielded 21.45 µg of total protein and 0.02 ng of ds-DNA per 1 × 10${}^{11}$ genome-containing particles (VG) respectively and a ratio of 46.9 total particles per VG. The second PCC run returned 20.8 µg of total protein and 0.02 ng of ds-DNA per 1 × 10${}^{11}$ genome-containing particles and a ratio of 56.5 total particles per VG.

## 4. Discussion

The main goal of this work was to assess the potential of using PCC for continuous AAV affinity capture; thus, three specific objectives were defined: (i) optimize elution recovery; (ii) model adsorption and guide PCC process development; and (iii) perform a continuous AAV affinity capture process using real feed conditions.

### 4.1. High Elution Recovery Yield Obtained after Buffer Screening

To optimize the elution buffer which maximizes AAV recovery yield, we used a full factorial DoE. The addition of arginine in combination with NaCl or CaCl${}_{2}$ has been shown to improve elution of proteins from affinity chromatography [25] media by preventing protein molecules from interacting with themselves or with other molecules, thus reducing aggregation [26]. By probing the design space using different concentrations of arginine and NaCl in the range of 0–500 mM, it was possible to improve the current elution recovery yield from an average of 55.5% (control) to an average of 89.8% (Figure 2). For the remaining chromatography experiments, the composition of the elution buffer was defined as 50 mM citrate, 500 mM of NaCl, and 350 mM of arginine, pH 3.5.

### 4.2. Modelling of the Breakthrough Curves Improves PCC Process Development

The generation of breakthrough curves is a critical step in batch process development, as column loading is usually dictated by a dynamic binding capacity. The analysis of breakthrough curves using a mathematical description of the adsorption process is a valuable tool that can be used for different goals. On the one hand, it allows for the prediction of the impact that certain operating conditions might have in the capture process; in the present case, AAV2 feed concentration and flow-rate were evaluated. On the other hand, the developed model allows for a deeper understanding of how operating conditions impact the PCC process, namely, the loading zone.

Although the usual strategy in a PCC process is to fully saturate the first column in the loading zone, this should always be balanced with eventual losses on the second column. These losses are related to the media capacity, other variables associated with the packing format, and dynamic variables such as flow rate and feed concentration. As seen in Figure 4, the PCC process begins to accumulate material when progressing through consecutive switching intervals. This transient state is particularly evident in the four initial switching intervals (*τ*_1_ to *τ*_4_) composing the first cycle. In this sense, the analysis of the concentration profile of the loading zone of a PCC process under steady state can potentially eliminate losses. In the present case, an arbitrary load volume of 200 CV was defined. From Figure 4, it can be observed that the combination of higher flow rates and high feed concentration levels results in AAV exiting the loading zone. In this sense, the model developed here was used to recalculate the outlet concentration of the loading zone under a defined load volume scenario (175 CV), using a feed concentration of 2 × 10${}^{12}$ TP mL${}^{-1}$ and a flow rate of 0.51 mL min${}^{-1}$. The new outlet concentration at the end of the first PCC cycle can be reduced to less than 1% using this scenario. The work proceeded with a load volume of 175 CV for the scenario involving high flow rates and feed concentration.

### 4.3. Continuous Affinity Capture Using 4C-PCC

The objective of the PCC runs we performed was to determine the consistency of the continuous capture process using real feed material. In order to ensure a minimum number of cycles (five) was performed with all feed materials, it was decided to perform the study using 0.2 mL pre-packed columns. This decision was mainly related to the capacity of the affinity medium we used and the expected throughput inherent in continuous processing systems. This resulted in a PCC process using four columns during six cycles. Additionally, due to the high UV signal found in the sample detector and no visible breakthrough of AAV found in the frontal experiments, the dynamic control capabilities of the ÄKTA pcc (as reported elsewhere [19,27,28]) were not used. In this sense, a time-based loading was performed.

Figure 5 reports the periodic UV${}_{280}$ signal of the recovery zone. Column wash was started with 25 CV of wash buffer (gray area) followed by 25 CV of elution buffer (light blue). A deeper look of the chromatograms in Supplementary Figure S2 reveals that the eluted peaks across the four columns are not identical, although a cyclic pattern can be observed over a period of four columns. This can be explained by the differences in packing across the four columns used. From the superimposition of the UV${}_{280}$ profiles (Figure 5A,B), it can be seen that, apart from the initial transient zone, the elution profiles are similar for each column. In fact, for the second PCC run (Figure 5B), the elution peak of column 3 is higher in comparison to the other elution peaks from the other columns. This periodic behaviour is expected due to the inherent cyclic procedures that all the columns are subject to within the process, and indicates that a stable cyclic steady state was achieved.

The characterization of the eluates of the two PCC runs performed is reported in Figure 6. The AAVs recovered from both processes show a similar morphology, as determined by TEM. Additionally, the main AAV capsid proteins are present in the Western blot analysis for the PCC runs. Figure 6B depicts a representative Western blot analysis of one of the batch runs performed during elution buffer optimization. All the samples show the same proportion of VP1, VP2, and VP3. Regarding virus recovery, as reported in Figure 6C, the observed difference can be explained by the modification made to the recovery procedure. The elution pool from run 1 shows a lower yield in comparison to the batch experiments performed (63.2% vs. 89.8%). It is hypothesized that this might be related to the dead volumes not accounted for in elution. In order to overcome this, the volume recovered in the elution fraction was doubled from 1.65 mL to 3.3 mL for run 2. With this modification, virus recovery could be increased from an average of 63.2% to 82.8%. This recovery value is in agreement with the optimized batch processes. Concerning the particle size distribution of the recovered AAV (Figure 6D,E), the differences observed (average diameter and polidispersity) in run 1 may be due to the presence of the larger population centered at 4173 nm, which, although evident in intensity weight analysis, is negligible when looking at the number-based distribution. Purity was analysed by TP/VG ratio in addition to ds-DNA and total protein per base of 1 × 10${}^{11}$ VG. Both runs returned similar values for the quantities mentioned. While these quantities might have a certain degree of dependence upon the feed material quality (especially for the TP/VG ratio), ds-DNA and total protein reduction are mostly associated with the selectivity provided by affinity chromatography. The last part of process characterization is relative to continuity. As seen in Figure 6F, continuous feeding takes place in the designed cycles of operation, as the duration of the elution and regeneration tasks are smaller than the switching interval imposed.

To assess productivity, a hypothetical batch process was compared against the developed PCC for the same level of bed saturation observed in Figure 4 for the end of the first operating cycle. This was accomplished by using the developed model to calculate the required volume to be injected (95 mL) in the batch process. Productivity was defined as the number of TP processed per unit of time per unit of chromatography medium. Additionally, for the batch process, the same PCC inlet concentration (run 2), flow-rates, and volumes were adopted for the different steps (wash, elution, and regeneration). The PCC process returns a productivity of 6.6 × 10${}^{14}$ TP mL${}^{-1}$ h${}^{-1}$ against 2.3 × 10${}^{14}$ TP mL${}^{-1}$ h${}^{-1}$, nearly a three-fold improvement. Although the same saturation level was considered for both processes, the simultaneous operation of the three zones (loading, recovery, and regeneration) and the accumulation of material from previous loading cycles enables improved media utilization.

## 5. Conclusions

Continuous affinity capture of AAV2 using PCC was proposed and developed in this study. The approach followed herein linked small-scale experimental studies to the optimization of the elution buffer and a mathematical model of AAV2 adsorption to Capto AVB. A four-column PCC operation was designed with minimal experimental effort to identify the implications of feed concentration and flow-rate in column adsorption behaviour. It was found that the continuous process achieved a cyclic steady state, as demonstrated by the periodic UV${}_{280}$ column elution profiles. The mathematical model showed an accumulation of material in the loading zone during the first cycle. This accumulation translates into higher saturation and utilization of the affinity chromatography medium as compared to a batch process. A productivity calculation based on processed AAV2 particles per unit volume of time and chromatography medium shows that the PCC process (run 2) outperforms batch processing by nearly three times. Both processes (batch and PCC) enabled high elution recovery yields (>82%), and the characteristic AAV capsid proteins were present in Western blot analysis. The characterization of the PCC runs show that the eluted viruses have a population size distribution centred around 26 nm, along with the typical icosahedral morphology.

This study demonstrates that minimal experimental effort is required to design a PCC process cycle and benefit from a continuous processing of AAV with higher productivities. Nevertheless, the true potential of using such technologies can only be fully realized by performing an integration with other continuous operating units (upstream and downstream of PCC). Moving from batch to continuous operating units, such as PCC will potentiate several opportunities for the reduction or removal of non-value-added equipment and unit operations in AAV processing. 

## Figures and Tables

**Table 1:** Model parameters obtained from the fitting of frontal experiments. Column dimensions (L × d [cm]): 1 × 0.5; bed porosity (ε): 0.4; extra-column volume: 1.6 mL.

Parameter	
Pe	26
*q*${}^{sat}$ (TP mL${}^{-1}$ )	$1.26\times 10^{15}$
*k*${}_{des}$ (TP mL${}^{-1}$ min${}^{-1}$)	$9.36\times 10^{7}$
$\frac{k_{des}}{k_{ads}}$ (TP mL${}^{-1}$ min${}^{-1}$)	$4.40\times 10^{-1}$

## Data Availability

All data are contained within the article.

## Supplementary Materials

The following supporting information can be downloaded at: https://www.mdpi.com/article/10.3390/pharmaceutics14071346/s1, Figure S1: Summary of fit from DoE; Figure S2: Chromatograms of PCC runs 1 and 2; Figure S3: Uncropped Transmission Electron Microscopy pictures; Table S1: Experimental matrix used in the DoE.

## References

[1] Pang J., Lauramore A., Deng W., Li Q., Doyle T.J., Chiodo V., Li J., Hauswirth W.W. (2008). Comparative analysis of in vivo and in vitro AAV vector transduction in the neonatal mouse retina: Effects of serotype and site of administration. Vis. Res..

[2] Penaud-Budloo M., François A., Clément N., Ayuso E. (2018). Pharmacology of recombinant adeno-associated virus production. Mol. Ther.-Methods Clin. Dev..

[3] Duan D. (2018). Systemic AAV micro-dystrophin gene therapy for Duchenne muscular dystrophy. Mol. Ther..

[4] Moleirinho M.G., Silva R.J., Alves P.M., Carrondo M.J., Peixoto C. (2020). Current challenges in biotherapeutic particles manufacturing. Expert Opin. Biol. Ther..

[5] Qu W., Wang M., Wu Y., Xu R. (2015). Scalable downstream strategies for purification of recombinant adeno-associated virus vectors in light of the properties. Curr. Pharm. Biotechnol..

[6] Adams B., Bak H., Tustian A.D. (2020). Moving from the bench towards a large scale, industrial platform process for adeno-associated viral vector purification. Biotechnol. Bioeng..

[7] Nass S.A., Mattingly M.A., Woodcock D.A., Burnham B.L., Ardinger J.A., Osmond S.E., Frederick A.M., Scaria A., Cheng S.H., O’Riordan C.R. (2018). Universal method for the purification of recombinant AAV vectors of differing serotypes. Mol. Ther.-Methods Clin. Dev..

[8] Kaludov N., Handelman B., Chiorini J.A. (2002). Scalable purification of adeno-associated virus type 2, 4, or 5 using ion-exchange chromatography. Hum. Gene Ther..

[9] Tomono T., Hirai Y., Okada H., Miyagawa Y., Adachi K., Sakamoto S., Kawano Y., Chono H., Mineno J., Ishii A. (2018). Highly efficient ultracentrifugation-free chromatographic purification of recombinant AAV serotype 9. Mol. Ther.-Methods Clin. Dev..

[10] Xie Y., Mun S., Kim J., Wang N.H.L. (2002). Standing wave design and experimental validation of a tandem simulated moving bed process for insulin purification. Biotechnol. Prog..

[11] Silva R.J., Rodrigues R.C., Osuna-Sanchez H., Bailly M., Valéry E., Mota J.P. (2010). A new multicolumn, open-loop process for center-cut separation by solvent-gradient chromatography. J. Chromatogr. A.

[12] Girard V., Hilbold N.J., Ng C.K., Pegon L., Chahim W., Rousset F., Monchois V. (2015). Large-scale monoclonal antibody purification by continuous chromatography, from process design to scale-up. J. Biotechnol..

[13] Müller-Späth T., Aumann L., Melter L., Ströhlein G., Morbidelli M. (2008). Chromatographic separation of three monoclonal antibody variants using multicolumn countercurrent solvent gradient purification (MCSGP). Biotechnol. Bioeng..

[14] Cristancho C.A.M., Seidel-Morgenstern A. (2016). Purification of single-chain antibody fragments exploiting pH-gradients in simulated moving bed chromatography. J. Chromatogr. A.

[15] Kröber T., Wolff M.W., Hundt B., Seidel-Morgenstern A., Reichl U. (2013). Continuous purification of influenza virus using simulated moving bed chromatography. J. Chromatogr. A.

[16] Nestola P., Silva R.J., Peixoto C., Alves P.M., Carrondo M.J., Mota J.P. (2015). Robust design of adenovirus purification by two-column, simulated moving-bed, size-exclusion chromatography. J. Biotechnol..

[17] Fischer L.M., Wolff M.W., Reichl U. (2018). Purification of cell culture-derived influenza A virus via continuous anion exchange chromatography on monoliths. Vaccine.

[18] Nestola P., Silva R.J., Peixoto C., Alves P.M., Carrondo M.J., Mota J.P. (2014). Adenovirus purification by two-column, size-exclusion, simulated countercurrent chromatography. J. Chromatogr. A.

[19] Mendes J.P., Silva R.J., Berg M., Mathiasson L., Peixoto C., Alves P.M., Carrondo M.J. (2021). Oncolytic virus purification with periodic counter-current chromatography. Biotechnol. Bioeng..

[20] Moleirinho M.G., Silva R.J., Carrondo M.J., Alves P.M., Peixoto C. (2019). Exosome-based therapeutics: Purification using semi-continuous multi-column chromatography. Sep. Purif. Technol..

[21] Schwarz H., Fons J.G., Isaksson M., Scheffel J., Andersson N., Andersson A., Castan A., Solbrand A., Hober S., Nilsson B. (2022). Integrated continuous biomanufacturing on pilot scale for acid-sensitive monoclonal antibodies. Biotechnol. Bioeng..

[22] Coolbaugh M.J., Varner C.T., Vetter T.A., Davenport E.K., Bouchard B., Fiadeiro M., Tugcu N., Walther J., Patil R., Brower K. (2021). Pilot-scale demonstration of an end-to-end integrated and continuous biomanufacturing process. Biotechnol. Bioeng..

[23] Scheffel J., Isaksson M., Gomis-Fons J., Schwarz H., Andersson N., Norén B., Solbrand A., Chotteau V., Hober S., Nilsson B. (2022). Design of an integrated continuous downstream process for acid-sensitive monoclonal antibodies based on a calcium-dependent Protein A ligand. J. Chromatogr..

[24] Guiochon G., Felinger A., Shirazi D.G. (2006). Fundamentals of Preparative and Nonlinear Chromatography.

[25] Chen S.W., Tan D., Yang Y.S., Zhang W. (2020). Investigation of the effect of salt additives in Protein L affinity chromatography for the purification of tandem single-chain variable fragment bispecific antibodies. mAbs.

[26] Arakawa T., Ejima D., Tsumoto K., Obeyama N., Tanaka Y., Kita Y., Timasheff S.N. (2007). Suppression of protein interactions by arginine: A proposed mechanism of the arginine effects. Biophys. Chem..

[27] Chmielowski R.A., Mathiasson L., Blom H., Go D., Ehring H., Khan H., Li H., Cutler C., Lacki K., Tugcu N. (2017). Definition and dynamic control of a continuous chromatography process independent of cell culture titer and impurities. J. Chromatogr. A.

[28] Gerstweiler L., Billakanti J., Bi J., Middelberg A.P. (2022). Control strategy for multi-column continuous periodic counter current chromatography subject to fluctuating inlet stream concentration. J. Chromatogr. A.

