# Supplementary material for: Continuous Affinity Purification of Adeno-Associated Virus Using Periodic Counter-Current Chromatography
Source: Pharmaceutics. 2022 Jun 25;14(7):1346. doi: 10.3390/pharmaceutics14071346 (PMC9323845; doi:10.3390/pharmaceutics14071346)
Supplement: Supplementary file 1 [file pharmaceutics-14-01346-s001.zip › pharmaceutics-1756598-supplementary.pdf]

CONTINUOUS AFFINITY PURIFICATION OF ADENO-ASSOCIATED VIRUS USING PERIODIC COUNTER-CURRENT CHROMATOGRAPHY

João P. Mendes[1,2] , Magnus Bergman[3], Anita Solbrand[3], Cristina Peixoto[1,2] , Manuel J. T. Carrondo[1,2] and Ricardo J. S. Silva[1,2,*]

[1]iBET, Instituto de Biologia Experimental e Tecnológica, Apartado 12, 2780-901 Oeiras, Portugal
[2]ITQB NOVA, Instituto de Tecnologia Química e Biológica António Xavier, Universidade Nova de Lisboa, Av. da República, 2780-157 Oeiras, Portugal
[3]Cytiva, 751 84 Uppsala, Sweden

Supplementary Table S1: Experimental matrix used in the design of experiments reported in Figure 2A.

Exp No.	Exp Name	Run Order	[NaCl] mM	[Arginine] mM
1	N1	2	0	0
2	N2	6	500	0
3	N3	1	0	500
4	N4	4	500	500
5	N5	3	250	250
6	N6	5	250	250
7	N7	7	250	250

Supplementary Figure S1

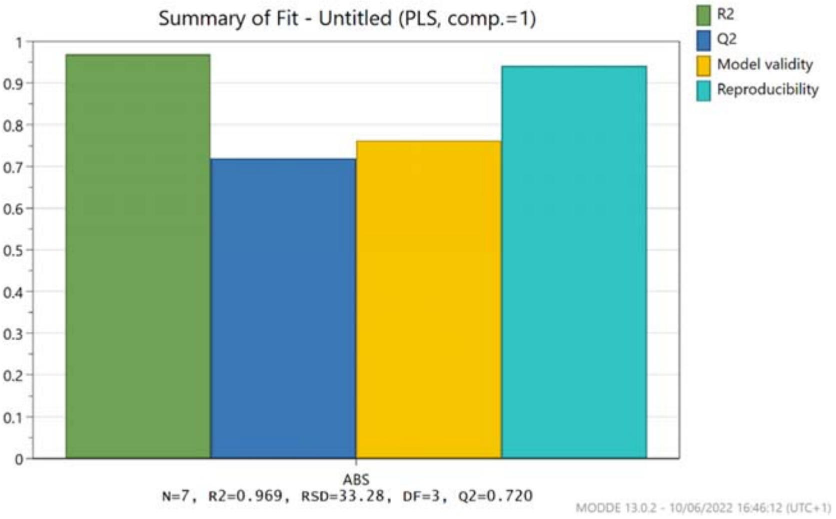

Supplementary Figure S1: Summary of fit from DoE reported in Figure 2.

Supplementary Figure S2

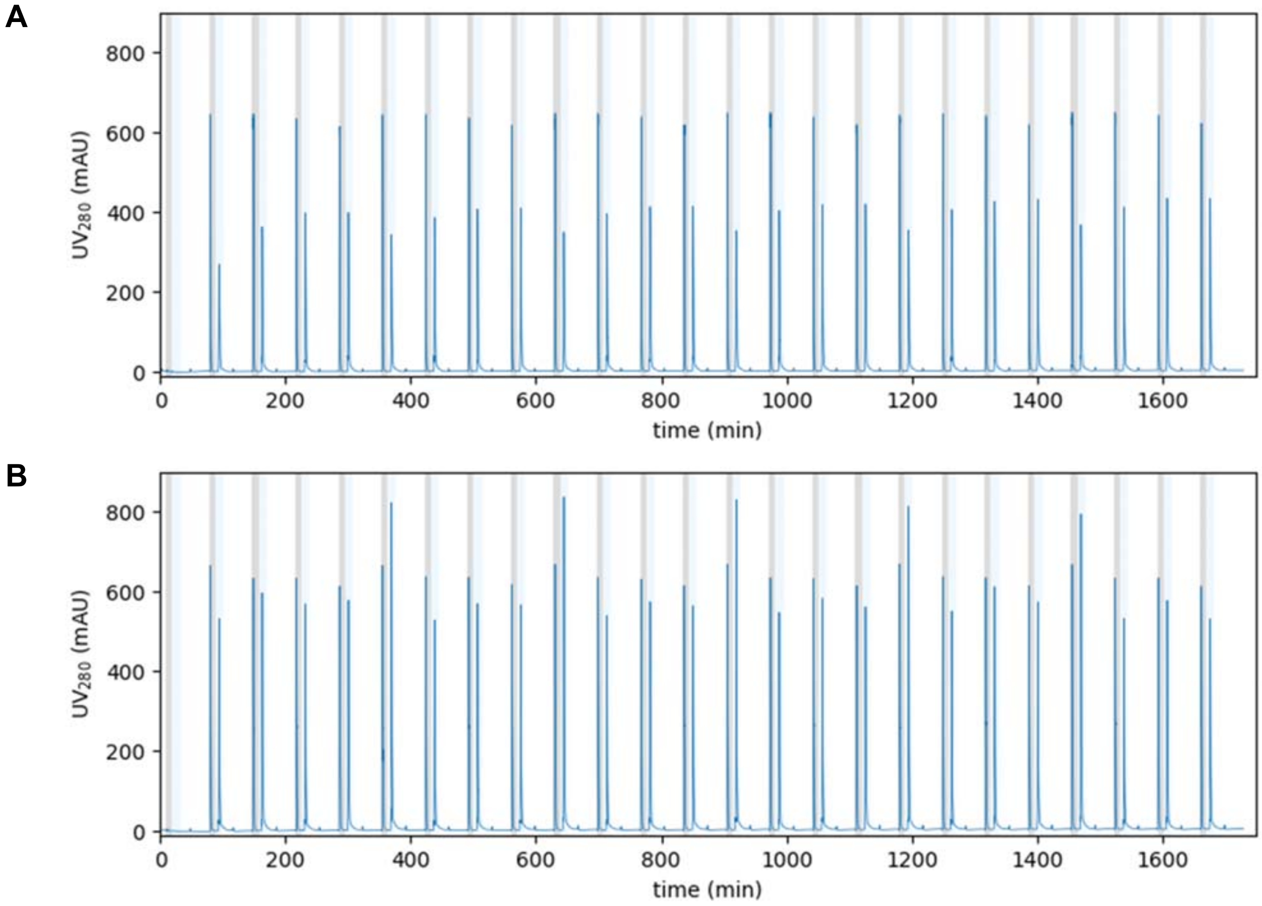

Supplementary Figure S2: Chromatograms of PCC runs 1 (A) and 2 (B); UV280 profiles obtained at the outlet of the elution zone over the 6 cycles performed in each run; gray zone marks column wash and light blue elution.

Supplementary Figure S3

A

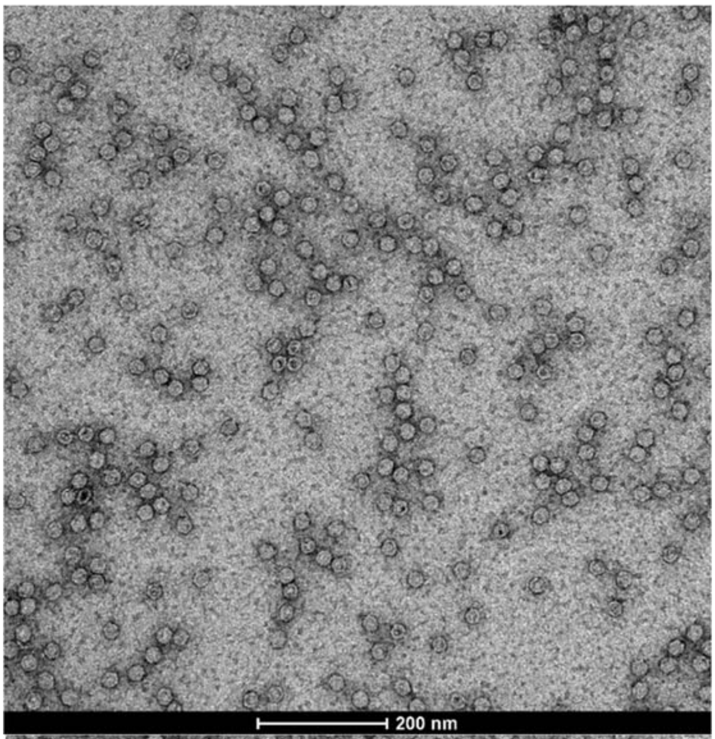

B

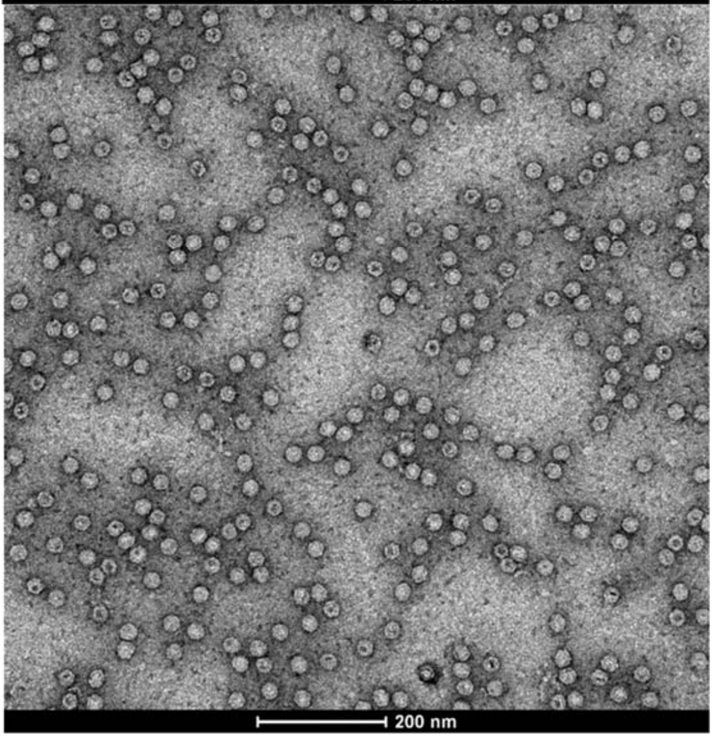

Supplementary Figure S3: Uncropped Transmission Electron Microscopy pictures. The figures were used for building Figures 6A and 6B (A and B respectively).
